# Pharmacokinetic Profile of Fentanyl in the Koala (*Phascolarctos cinereus*) after Intravenous Administration, and Absorption via a Transdermal Patch

**DOI:** 10.3390/ani11123550

**Published:** 2021-12-14

**Authors:** Fumie Tokonami, Benjamin Kimble, Merran Govendir

**Affiliations:** 1 Currumbin Wildlife Hospital, Currumbin, Gold Coast 4223, Australia; ftokonami@cws.org.au; 2 Sydney School of Veterinary Science, The University of Sydney, Camperdown, Sydney 2006, Australia; benjamin.kimble@sydney.edu.au

**Keywords:** fentanyl, koala, *Phascolarctos cinereus*, transdermal patch, analgesia, opioid

## Abstract

**Simple Summary:**

Koalas can be injured by cars and bushfires, and be affected by painful infectious diseases. When koalas undergo surgery to repair broken bones, they require analgesia. Fentanyl is a potent opioid that can be administered during surgery to provide analgesia. This study describes the rate of elimination of fentanyl in koalas’ blood when administered as a single intravenous injection and consequently calculates the dose rate to administer a constant rate fentanyl infusion into the koalas’ veins to provide short-term pain control. Fentanyl can also be absorbed via the skin into the circulation when applied as a transdermal patch. Although the data for transdermal fentanyl patch absorption is from two koalas only, the results demonstrate that when a patch is applied, pain control is likely to occur 12 h after application to koalas’ skin. Fentanyl may provide effective pain control to koalas either as an intravenous infusion or as a transdermal patch.

**Abstract:**

Fentanyl was administered as a single intravenous bolus injection at 5 µg/kg to five koalas and fentanyl plasma concentrations for a minimum of 2 h were quantified by an enzyme-linked immunosorbent assay (ELISA). The median (range) fentanyl elimination half-life and clearance were 0.53 (0.38–0.91) h, and 10.01 (7.03–11.69) L/kg/h, respectively. Assuming an analgesic therapeutic plasma concentration of 0.23 ng/mL (extrapolated from human studies), an intravenous constant infusion rate was estimated at approximately between 1.7 to 2.7 µg/kg/h (using the clearance 95% confidence intervals). A transdermal fentanyl patch was applied to the antebrachium of an additional two koalas for 72 h. Fentanyl plasma concentrations were determined during the patch application and after patch removal at 80 h. The fentanyl plasma concentration was greater than 0.23 ng/mL after 12 to 16 h. While the patch was applied, the maximum fentanyl concentration was approximately 0.7 ng/mL from 32 to 72 h. Fentanyl plasma concentrations increased to 0.89 ng/mL 1 h after the patch was removed, and then decreased to a mean of 0.47 ng/mL at 80 h. The transdermal fentanyl patch is likely to provide some level of analgesia but should be initially co-administered with another faster acting analgesic for the first 12 h.

## 1. Introduction

Wild koalas may be injured by car strikes, bushfires or animal attacks, potentially requiring analgesia to improve the quality of patient recuperation and survival. There are only two published pharmacokinetic (PK) profiles for analgesics for the koala. The first study investigated the PK profile for the non-steroidal anti-inflammatory drug (NSAID) meloxicam which was found to have a short elimination half-life of 1.19 h (range 0.71 to 1.62 h) [1,2], compared with 24 h in dogs [3] and approximately 13 h in humans [4]. Therefore, in contrast to many other species, meloxicam would require frequent dosing during a 24-h period for koalas which is problematic for a wild, non-domesticated species. Recently the PK profile of tramadol as a 4 mg/kg subcutaneous injection in the koala has been published and concluded that when injected twice daily, this dosing regimen should provide some analgesia [5]. However, the lack of the description of the analgesic PK profiles for koalas highlights the requirement to identify analgesics that are not only efficacious, but also have a longer duration of analgesia to minimise patient handling.

Full agonists at µ (mu) opioid receptors are recognised to provide strong analgesia in humans and animals [6]. The PK profile of opioid-receptor agonists, such as fentanyl, have not been reported in any Australian wildlife species, yet they have been used as analgesics to treat wildlife species, including koalas [7,8,9]. Fentanyl is one of the most potent of the µ receptor full agonists [6] and all formulations are legislated as Schedule 8/‘Controlled drug’ throughout Australia. It is available in a variety of formulations including an injectable formulation and as a transdermal patch. The aim of this study was to describe the PK profile of fentanyl citrate after a single intravenous (i.v.) bolus. Some information was also obtained on the PK profile of the transdermal fentanyl patch (TFP) when applied to two koalas.

## 2. Materials and Methods

### 2.1. Animals and Housing

The University of Sydney Animal Ethics Committee approved this study (protocol numbers N00/2-2012/3/5653 and 2015/877).

Clinically normal, adult koalas (*n* = 8; 5 males, 3 females), ranging in age from 3 to 8 years, as determined by tooth wear [10], were recruited opportunistically from those housed temporarily at Currumbin Wildlife Hospital (CWH), Currumbin, Queensland, Australia. The koalas were selected based on being mature (>2 years), without a dependent joey and not receiving any medications. Koalas had initially been admitted to the hospital for injuries caused by a vehicle strike (*n* = 3), chlamydial conjunctivitis (*n* = 2) or presented for relocation after being found in an unsuitable habitat (*n* = 3). All koalas were assessed as clinically normal based on a physical examination conducted under general anaesthesia by veterinarians at CWH and consisting of thoracic and abdominal radiographs, ultrasound, abdominocentesis and bone marrow cytology. The two koalas that had chlamydial conjunctivitis (koalas K2 and K4) had undergone anti-chlamydial treatment involving a 28-day course of chloramphenicol injections administered subcutaneously once daily (60 mg/kg) as well as chloramphenicol eye ointment applied twice daily [11]. They received their last chloramphenicol injections two weeks prior and were both negative on post-treatment polymerase chain reaction (PCR) testing for chlamydial infection and had no signs of clinical disease. The koalas were being prepared for release and were not on any supplemental feeds during this study. Their median weight was 6.1 kg (weight range was 4.2 to 9.7 kg). Information on the individual koala’s sex, weight and approximate age are provided in supplementary Table S1. Their haematology and biochemistry analytes were within the ‘clinically normal’ reference ranges [12,13]. The koalas were housed in standard veterinary hospital enclosures and supplied with food ad libitum (various *Eucalyptus* spp.) and water throughout the study.

In order to facilitate placement of a 20-gauge i.v. catheter into cephalic veins for serial blood sampling (both right and left veins for the i.v. group and left vein only for the TFP group), koalas were administered an intramuscular injection of alfaxalone (Alfaxan; Jurox, Rutherford, Australia), 2 to 3 mg/kg prior to mask induction using 5% isoflurane in 100% oxygen delivered by a precision vaporizer. Catheters were held in place using cohesive bandage (Rip Rap^®^, ZebraVet, Rocklea, Australia). Heparinised saline (5 IU/mL) at 0.2 mL was used to flush the catheter before and after blood collection through the injection port.

### 2.2. Drug Administration and Blood Collection

#### 2.2.1. Fentanyl Intravenous Administration

For the i.v. group, six koalas were administered fentanyl citrate (DBL^®^ Fentanyl Injection; Pfizer Australia, Sydney, Australia) as a single injection via an injection port on the intravenous catheter placed in the right cephalic vein approximately 30 min after intramuscular (i.m.) injection of alphaxalone. Animals were awake when fentanyl was injected, but still sedated from alphaxalone.

The first koala was injected with 10 µg/kg; however, this resulted in excessive sedative and cardio-respiratory depression effects and therefore the next five koalas were medicated at 5 µg/kg. The volume of fentanyl injection administered was 0.6 to 1.2 mL depending on the weight of the koala and was injected over 10 s. Naloxone (DBL^®^ Naloxone Hydrochloride Injection; Pfizer Australia, Sydney, Australia) 400 µg/mL was prepared as an injectable rescue antidote in case any koala developed significant respiratory depression or any other adverse effects. Heart rate as beats per minute (bpm) and respiratory rate per minute (RR) were monitored by chest auscultation using a stethoscope, and oxygen saturation (SPO_2_%) was monitored using a pulse oximeter (Life Sense IPX2, Nonin Medical, Inc., Plymouth, MN, USA) for each animal over the first 60 min of the fentanyl i.v. administration.

Blood collection was undertaken via the i.v. catheter placed in the left cephalic vein throughout the first 24 h of the study, unless a problem was experienced collecting blood through this catheter in which case, the catheter in the right cephalic vein was used.

#### 2.2.2. Transdermal Fentanyl Patch (TFP) Application

For TFP group, fentanyl patches (Fentanyl Sandoz^®^; Sandoz, Macquarie Park, Australia) with a delivery rate of 25 µg/h were adhered to clipped, cleaned, and dried skin on the medial aspect of the right antebrachium in two additional koalas. Fentanyl patches were removed at 72 h. Objective assessment of demeanour and mobility of koalas at the time of blood collection were recorded. Due to the transdermal patch being applied for more than three days, appetite score (0 = no food eaten, 1 = one-eighth of food offered eaten, 2 = one-quarter of food offered eaten, 3 = half of food offered eaten), faecal pellet count and weight were recorded as part of the daily observation of these animals.

#### 2.2.3. Blood Collection

Serial blood samples (up to 1.3 mL) for fentanyl concentration determination were collected into lithium heparin tubes at the following time points after i.v. fentanyl administration: time (t) = 0, 2, 5, 10, 15, 30 min and then 1, 2, 4, 6, 8 and 24 h and after TFP administration: t = 0, 1, 2, 4, 8, 24, 32, 48, 56, 72, 73, 74, 76 and 80 h.

Additional blood samples (2 mL) were collected in ethylenediaminetetraacetic acid (EDTA) and in a clot tube from both groups at t = 0 h to determine baseline haematology and biochemistry values, respectively; and at t = 24 h after i.v. administration. In order to separate the plasma or serum, blood samples were centrifuged at 6000 rpm for 10 min within 1 h of collection. The resulting plasma or serum were transferred into plain tubes. Plasma samples for fentanyl quantification were stored at −20 °C and protected from light until analysis.

### 2.3. Fentanyl Assay

Plasma concentrations of fentanyl were determined using a commercially available enzyme-linked immunoassay (ELISA) kit, the Abnova Fentanyl (Human) ELISA Kit KA0931 Lot EK13959 (Sapphire Bioscience, Redfern, Australia) which consisted of a 96-well polystyrene micro-titre plate coated with purified polyclonal anti-fentanyl, horseradish peroxidase labelled fentanyl conjugate, fentanyl 5 ng/mL in human urine as a positive standard, negative standards, tetra-methylbenzidine (TMB) substrate, and a stop reagent. The instrumentation used for reading the ELISA plate was a BioTek Microplate Reader with Gen5 software (Agilent Technologies, Winooski, VT, USA).

Standards, controls, and koala plasma samples (all 20 µL) were added each into duplicate wells. Horseradish peroxidase labelled with fentanyl derivative conjugate was added to each well (100 µL). The plate was then incubated in the dark at room temperature (18 to 26 °C) for 60 min. The wells were then washed thoroughly, and 100 µL of TMB substrate reagent was added to each well. After further incubation in the dark at room temperature (18 to 26 °C) for 60 min, 100 µL of the stop reagent was added to each well and the plate was read at 450 nm. The mean absorbance of each duplicate was used to determine each sample’s fentanyl concentration.

A standard curve to read the fentanyl concentrations in the plasma samples, was generated on two separate occasions. On the first occasion the standard absorbances of the known fentanyl concentrations were generated on the same plate with the plasma samples from the i.v. fentanyl administration. There were five standards of known fentanyl concentration: 0, 0.1, 0.5, 1 and 5 ng/mL that were incorporated into the ELISA and run with all samples. The mean absorbance of the zero standard was subtracted from the mean absorbance of the other standards. The log of mean absorbance was plotted against log of the known concentration and the equation of this regression line generated. The accuracy of the 0.1, 0.5, 1 and 5 ng/mL standards’ concentration was then ‘back calculated’ from the standard curve; this value was converted from log to the linear scale to give the final concentration. Each of these standard concentrations were checked to ensure they had an accuracy of >80%. Thus 0.1 ng/mL was considered the lower limit of quantification (LLOQ) and 5 ng/mL was the highest limit of quantification (HLOQ). Any values outside this range were considered inaccurate.

On the second occasion the standard absorbances of the known fentanyl concentrations were generated with the plasma samples from the TFP administration and one i.v. fentanyl curve (i.e., repetition of koala K6′s plasma samples). The standard curve was generated as for the first. Concentrations between 0.1 to 1 ng/mL could be determined with 90% accuracy.

### 2.4. Pharmacokinetic Analysis

Standard pharmacokinetic parameters and indices for i.v. administration were generated as a non-compartment model using PKSolver [14].

The peak plasma concentration (C_max_) and time to reach C_max_ (i.e., T_max_) were determined by visual inspection of the time vs. concentration curve. The elimination constant (k_el_) was estimated by semi-log linear regression of slope of the elimination phase, and elimination half-life (t_1/2_) was estimated by ln2/k_el_. Area under the curve (AUC_0–t_) and area under the first moment curves (AUMC_0–t_) from 0 to last observed concentration that was greater than the LLOQ (0.1 ng/mL) were determined by the linear trapezoidal method. The terminal segment of AUC_0–infinity (inf)_, i.e., AUC_t–inf_ was calculated as C_last_/k_el_, where C_last_ is the last measured plasma concentration and k_el_ is the slope during the terminal phase, as AUC_0–inf_ = AUC_0–last_ + AUC_last-inf_.

The AUMC was calculated as the sum of each of the concentrations multiplied by their time-point (h). The AUC and AUMC from the observed concentration to infinity were determined by Equation (1). The volume of distribution at pseudodistribution equilibrium (Vd_area_)_,_ volume of distribution at steady state (Vd_ss_) and clearance (Cl) were calculated by Equations (3) and (4) [15] and Equation (5) [16], respectively.

AUMC_t–inf_ = ((C_last_ × t_last_)/k_el_) + (C_last_/k_el_^2^) (1)

The volume of distribution: Vd_0_ = total dose/extrapolated concentration at t = 0 h (2)

Vd_area_ = (dose/AUC) × (1/k_el_) (3)

Volume of distribution at steady state: Vd_ss_ = (dose × AUMC)/(AUC^2^) (4)

Clearance (Cl) = k_el_ × Vd_area_ (5)

Mean residence time (MRT) = AUMC/AUC(6)

All PK parameters and indices are provided as median (range) and as mean ± standard deviation.

The dosage for a constant rate infusion = the desired plasma concentration *×* clearance [17] using the 95% confidence intervals of the mean clearance.

### 2.5. Statistical Analyses

The change in heart rate prior to, and over 30 min after fentanyl 5 µg/kg i.v. administration underwent a mixed-effects analysis using GraphPad Prism 9.0. (GraphPad, San Diego, CA, USA). The data also underwent a Tukey’s multiple comparisons test. The level of significance was accepted at *p* < 0.05.

## 3. Results

**Figure 1:**
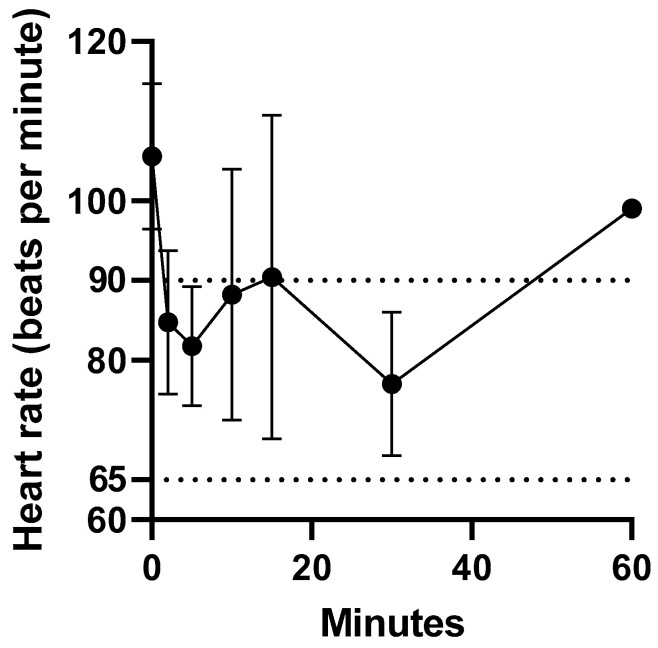
Mean ± S.D heart rate on administration of fentanyl 5 µg/kg as a single i.v. bolus over the first hour. Normal koala heart rate (bpm) is within dotted lines. There was only a significant difference in mean heart rate between 0 and 2 min (*p* = 0.04).

Intravenous administration of fentanyl citrate was well tolerated by all koalas, with no signs of pain or irritation observed. The heart rate of the koalas at t = 0 min ranged from 94 to 119 bpm. The heart rate reduction within 2 min of intravenous administration of fentanyl ranged from 13 to 34 %. However, the heart rate remained above 60 bpm (normal range 65 to 90 bpm [18]). There was only a significant difference in mean heart rate between 0 and 2 min (*p* = 0.04). The change in heart rate prior to and over 1 h after fentanyl administration is illustrated in Figure 1. The change in heart rate (bpm) after fentanyl administered as a single intravenous injection data for koalas (K1 to K5) is provided in supplementary Table S2.

Initially, one koala was administered 10 µg/kg i.v. and became bradypnoeic, i.e., 2 breaths per minute by 10 min (normal respiratory rate at rest is 10 to 15 bpm [19]). However, no intervention or reversal was carried out as pulse strength and SPO_2_ remained stable. The respiratory rate improved 15 min post fentanyl administration, and the koala was able to climb and sit in a tree after 1 h and thereafter. However, the dose was reduced to 5 µg/kg when administered to the other five koalas. All koalas maintained their peripheral arterial oxygen saturation (SPO_2_) between 97–100%. The data for the RR and SPO_2_ were incomplete and did not undergo any further analysis.

### 3.1. Pharmacokinetic Profile of Fentanyl after a Single i.v. Bolus Administration

**Figure 2:**
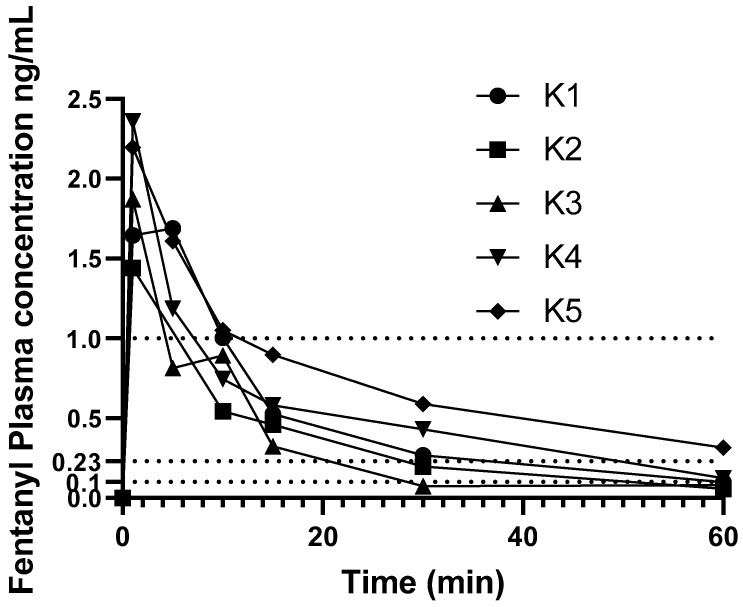
Change in fentanyl plasma concentration over time in minutes. Koala 1 dosed at 10 μg/kg the other five koalas were dosed at 5 µg/kg. Dotted lines at 0.23 and 1.0 ng/mL denote suggested analgesic range (see Discussion). Additional dotted line at 0.1 ng/mL denotes assay LLOQ.

Individual change in fentanyl concentrations over time are shown in Figure 2. Results from one koala (K6) administered fentanyl 5 µg/kg were excluded from pharmacokinetic analysis as this koala’s plasma fentanyl concentrations were above the higher limit of the assay (>5 ng/mL), and at some time-points did not decrease with time, thus this koala’s plasma fentanyl concentrations were omitted from PK calculations.

None of the koalas had detectable fentanyl in plasma at time zero. The PK parameters and indices following administration of a single i.v. bolus dose of fentanyl are provided in Table 1. Individual variation of time vs. concentration data was small in the first 15 min then became larger during the rest of the course of the study. The plasma fentanyl concentration was below 1 ng/mL in one koala at 5 min and in the others by 15 min as illustrated in Figure 2. The plasma fentanyl concentrations were below 0.2 ng/mL in the koalas dosed with 5 µg/kg by 30 min and in the koala dosed with 10 µg/kg, shortly after 1 h. The plasma fentanyl concentrations were below the limit of quantification (0.1 ng/mL) in three koalas at 1 h. The fentanyl plasma concentrations over time of koalas K1 to K6 are provided in supplementary Table S3. 

Constant infusion rate = desired plasma concentration (0.23 ng/mL) × Cl (within the 95% confidence intervals based on mean Cl (7.30–11.7 L/kg/h)) = 1.7 to 2.7 µg/h/kg.

### 3.2. Transdermal Administration of Fentanyl

Application of fentanyl patches was well tolerated by both koalas. One koala was seen gnawing at the bandage occasionally over the first 2 h, but it only lasted a few seconds each time, and the bandage was not penetrated or damaged. The actual TFP dosages ranged from 4.96 to 5.98 µg/kg/h as the same fentanyl patch (25 µg/h) was applied to the two koalas irrespective of difference in body weight.

**Figure 3:**
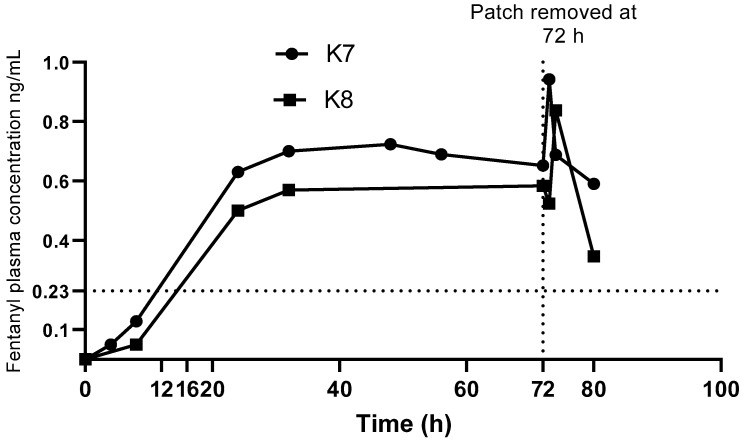
Fentanyl concentration vs time after transdermal fentanyl patch (TFP), (with a delivery rate of 25 µg/h), application and removal for both koalas. Dotted lines at 0.23 ng/mL denote suggested minimal analgesic range in human patient controlled analgesia models (see Discussion).

The change in plasma fentanyl concentrations over time on application and removal of the patch are shown in Figure 3. After application of the TFP, plasma fentanyl concentration was first detected at 4 h in one koala and 8 h in the other. Mean C_max_ of 0.89 ng/mL (range 0.84–0.94 ng/mL) was detected at 73 to 74 h.

## 4. Discussion

The median (range) fentanyl half-life in the koala 0.53 (0.38–0.91) h was similar to that of dogs of 0.76 h [20] but less than that reported in cats of 2.35 to 2.52 h [21] or 2.52 h [22]; or 1.2 h [23] to 2.58 h in goats [24]; or 1 h in horses [25]; or 1.2 h in alpacas [26]. It has been noted that some therapeutic drugs, such as the non-steroidal anti-inflammatory drug meloxicam, has a much shorter half-life in koalas than cats and dogs and other domesticated animals [1,11]; however, there does not seem to be obvious differences in the fentanyl elimination half-life in koalas compared to the other species.

The first koala in the i.v. group received an injection of fentanyl at 10 µg/kg. This dosage was safely used in dogs [20] and cats [22]. However, this dosage resulted in profound sedation, bradycardia and bradypnea in this koala leading to a halving of the administered dosage to the other koalas.

The reason for the anomaly in the fentanyl plasma concentrations of K6 that received a 5 µg/kg i.v. fentanyl bolus (results not provided in Table S2) remains unclear. This koala’s plasma samples underwent the ELISA on two occasions and on both provided equivocal results. The ELISA returned very high fentanyl concentrations for these plasma samples. Furthermore, some did not reduce in concentration with time and consequently, were excluded from PK calculations. This koala did not receive chloramphenicol treatment prior to fentanyl administration and although chloramphenicol is a known cytochrome P450 substrate inhibitor [27], it cannot be attributable for the fentanyl plasma concentrations abnormalities obtained from this koala. An ELISA to detect fentanyl plasma concentrations has been used in other animal studies due to its convenience [28,29], however another method such as liquid chromatography-mass spectrometry may provide greater accuracy and sensitivity [22,25].

All koalas experienced varying degrees of reduction in heart rate immediately after intravenous administration of fentanyl. In other studies involving a single i.v. administration of fentanyl at 2.5 µg/kg in goats [23] and at 4 µg/kg in horses [25], no significant change in heart rate was observed. Cardiovascular effects of fentanyl have been studied in laboratory animals [30,31]. In rabbits, administration of i.v. fentanyl significantly decreased the diameter and mean volumetric flow of the abdominal aorta, end-diastolic blood flow velocity of the left common carotid artery, mean arterial pressure, heart rate and body temperature [30]. In contrast, cardioprotective effects without haemodynamic effects following i.v. administration of fentanyl has been reported in an experimental model of myocardial ischemia in anesthetised rabbits [31]. In this study, fentanyl was administered i.v. while the koalas were not fully recovered from the sedative effect of alphaxalone administered i.m. approximately 30 min prior, in order to minimise handling stress of frequent blood collections during the first hour. The PK profile of alphaxalone has not been investigated in koalas, but the elimination half-life in cats and dogs is approximately 45 and 34 min, respectively [32,33]. Therefore, the decrease in heart rate during the first hour of the i.v. fentanyl administration may, in part, have also been influenced by the residual effect of alphaxalone.

There was no apparent change in the weight and appetite reported in koalas for the duration of this study. However, all six koalas in i.v. group exhibited increased sensitivity or resistance to handling after repeated blood collection, resulting in failure to collect blood samples at scheduled time points and/or termination of the study as early as 4 h after fentanyl administration in some animals. All koalas were from the wild, and had varying tolerance to handling prior to the study. They were destined to be released back into the wild after the study, therefore the negative change in their behaviour (such as vocalizing, ear flicking, moving away from and/or attempting to scratch or bite the blood collector) did not affect the outcome of rehabilitation. However, such observations need to be taken into consideration when designing further studies, especially if longer duration of serial blood sampling is required or involves animals in captive collections.

The TFP appeared to provide relatively constant plasma concentration of fentanyl for prolonged period in the two koalas, which is a desirable characteristic of the transdermal administration route. However, the mean C_max_ for the 25 µg/h TFP (0.73 ng/mL) was lower in the koalas than for cats with the same 25 µg/h patch (C_max_ for the cats ranged from 3.7 ± 1.6 to 6.1 ± 3.0 ng/mL [34]). Fentanyl is recognized to have superior transdermal absorption when the skin is warmer [35]. The lower C_max_ in the koala may be due to their slightly lower normal body temperature associated with marsupials of 36.5 °C [36].

An increase in fentanyl concentrations were observed 1 to 2 h after removal of the patch in the two koalas. Similar observations have been reported in dogs [28,37]. On removal of the patch there may be a redistribution of fentanyl between body compartments resulting in a rapid increase in plasma fentanyl concentration at removal of the patch followed by a gradual reduction as illustrated in Figure 3.

The plasma concentration required for optimal analgesia in koala was not determined. It has been suggested that a minimum plasma concentration of fentanyl required to provide analgesia was 1.07 ng/mL in a thermal threshold model in cats [38]. The minimum analgesic plasma concentrations determined by patient controlled analgesia (PCA) in response to abdominal incisions in humans is 0.23 ng/mL [39] or 0.63–1.54 ng/mL [40]. In the study of isoflurane-sparing effect of fentanyl in horses, high plasma concentrations of 16 ng/mL [41] and 8.43 ng/mL [42] failed to reduce the minimum alveolar concentration of isoflurane, although in the same study a plasma fentanyl concentration at 13.31 ng/mL resulted in an 18% reduction of mean alveolar concentration (MAC) [42]. In the absence of objective anti-nociceptive scoring or testing, a plasma fentanyl concentration between 0.23 to 1.0 ng/mL, extrapolated from studies in humans [43] and domestic animals [38], were used as a hypothetical target analgesic concentrations for the purpose of this study. Fentanyl can be used as a constant rate i.v. infusion for acute pain and this study does establish an approximate dose rate range of 1.7 to 2.7 µg/kg/h for koalas which is similar to that suggested from clinical observations of 3.0 µg/kg/h [7].

The results reported here do not support the use of TFP as the sole analgesic in koalas as the higher hypothetical target plasma fentanyl concentration of 1.0 ng/mL was not attained. A study with dogs [28] used 0.23 ng/mL as the minimum fentanyl concentration required to provide analgesia, which was a lower end of the minimum effective concentration range described in human studies [39]. If koalas only require plasma fentanyl concentration of 0.23 ng/mL to have analgesic effects, both koalas achieved this concentration 24 h after application of the TFP, and it was maintained to the last blood collection 8 h after removal of the patch. A reduced response to external stimuli such as manual restraint for blood sample collection, was observed 4 h post TFP administration, at which time the plasma fentanyl concentrations were less than 0.05 ng/mL. This perhaps suggests that a sedative effect was seen in the two koalas with plasma fentanyl concentrations much lower than required for provision of analgesia. Recruitment of more koalas for this study, more frequent blood collection especially during the first 24 h, longer duration of the patch placement and blood sampling up to, or beyond 24 h after patch removal would improve the description of the PK profile of the TFP for the koala. During the devastating Australian bush-fire season 2019/2020 the first author (FT) applied a TFP to many surviving burnt koalas to provide some long-lasting analgesia with some good clinical outcomes. This author also administers methadone initially for analgesia until significant fentanyl has been absorbed >0.23 ng/mL at approximately 12 to 16 h after patch application.

The application site for TFP in this study was chosen based on relative ease of securing the patch using a bandage wrapped on a limb compared to other areas of the body such as thorax or abdomen. In vitro studies on regional differences of the penetration of fentanyl through equine skin showed significantly lower penetration through the leg than thorax or inguinal regions [44]. This suggests that the plasma fentanyl concentrations observed in this study may be significantly lower than what could have been achieved if the TFP had been applied to the thorax or inguinal regions. An in vitro study into TFP absorption of fentanyl using skin samples harvested from different regions of koala bodies may be useful in determining the best site for application of the TFP.

There were several limitations to this study. Sample sizes were limited due to the rarity of admission of ’clinically normal’ koalas to the wildlife hospital. Even when there was an increase in the admission of clinically normal koalas during the start of their breeding season in August, it was not feasible to hold a releasable koala in care to carry out clinical trials over spring to summer due to lack of resources and housing spaces. For the same reasons it was not possible to have control groups involving the use of placebo, for example an injection of saline for i.v. group or a placement of hydrocolloidal wound dressing for TFP group. Comparison of PK indices in males and females was not possible in the studied population as there was only one female out of six animals in the i.v. group. Low number of females recruited to the study was largely due to exclusion of females with dependent joeys. A prolonged half-life and wider volume of fentanyl has been reported in females compared to male alpacas [26]. Further studies using equal number of male and female koalas are needed to demonstrate whether such observations occurs in koalas.

This study did not investigate whether the fentanyl administration reduced pain and there was no investigation to quantify the plasma therapeutic concentration of fentanyl required for analgesia in the koala. Development of a pain scoring system or facial grimace score may be useful in investigating the analgesic efficacy and determining the target concentration of fentanyl and other opioids and/or NSAIDs. Pharmacodynamic studies using an objective measurement of nociception such as thermal or mechanical threshold testing would be desirable, although validation of the method may be difficult as wild animals may experience stress simply by having humans in their proximity, when physical contact is initiated or when restrained [7].

## 5. Conclusions

A single i.v. bolus dose of fentanyl at 10 µg/kg is likely to result in adverse effects (likely temporary) in the koala. However, koalas seem to tolerate a single bolus dose of 5 µg/kg. The half-life of a single i.v. bolus is approximately 0.6 h = 36 min, and is likely too short to have any significant analgesic effects. However, a constant rate infusion dose could be calculated as approximately 1.7 to 2.7 µg/kg/h. Although the data provided is limited, this study provides some information on the PK profile of the TFP when applied to the antebrachium of koalas. The limited data suggests that plasma fentanyl concentrations that exceed 0.23 ng/mL are maintained from 12 to 72 h after the patch is applied.

## Figures and Tables

**Table 1:** Pharmacokinetic parameters and indices calculated from a non-compartment analysis following administration of a single i.v. 5 mg/kg bolus dose of fentanyl.

Pharmacokinetic Parameters and Indices	Median (Range)	Mean ± SD	Number of Observations
k_el_ (1/h)	1.30 (0.76–1.83)	1.25 ± 0.40	5 *
t_1/2_ (h)	0.53 (0.38–0.91)	0.60 ± 0.20	5 *
C_max_ (ng/mL)	1.87 (1.44–2.36)	1.84 ± 0.39	4
Plasma concentration at t = 2 min (ng/mL)	2.62 (1.65–3.48)	2.48 ± 0.90	4
AUC_0–t_ (ng/mL) × h	0.60 (0.41–1.16)	0.54 ± 0.14	4
AUC _t–inf_ (ng/mL) × h	0.14 (0.09–0.44)	0.19 ± 0.16	4
AUC_0–inf_ (ng/mL) × h	0.60 (0.43–1.17)	0.56 ± 0.18	4
AUC_0–t_ /AUC_0-inf_ (%)	100 (0.96–100)	99 ± 2	4
AUMC_0–inf_ (ng/mL) × h^2^	0.28 (0.17–1.04)	0.25 ± 0.09	4
Mean residence time (MRT) (h)	0.47 (0.40–0.89)	0.54 ± 0.20	5 *
Vd (L); (L/kg)	20.87 (9.05–26.01)	18.96 ± 6.58; (2.55 ± 0.99)	4
Vd_area_ (L); (L/kg)	63.81 (33.28–68.73); (7.20 (5.28–8.97))	52.5 ± 18.36; (6.80 ± 1.64)	4
Vd_ss_ (L); (L/kg)	35.11 (22.71–49.06); (4.32 (3.61–5.060))	32.46 ± 11.09; (4.19 ± 0.66)	4
Cl (L/h); (L/kg/h)	71.62 (44.30–113.10); (10.01 (7.03–11.69))	68.1 ± 34.90; (8.57 ± 3.22)	4
95% confidence interval for clearance mean (L/kg/h)	NA	7.3–11.7	5

k_el_: elimination constant; t_1/2_: elimination half-life; C_max_: maximum concentration; AUC: area under the curve; AUMC: area under the moment curve, MRT: mean residence time; Vd: volume of distribution extrapolated to t = 0 h. Vd_area_: volume of distribution at pseudodistribution equilibrium; Vd_ss_: volume of distribution at steady state; Cl: clearance. As K1 was dosed at 10 µg/kg and the other four koalas were dosed at 5 µg/kg: * indice independent of dose therefore these indices could be calculated for five koalas. Other indices are dependent on dose and therefore calculated on the four koalas dosed with 5 µg/kg; NA = not applicable.

## Data Availability

Data generated by this study are available in this manuscript and in the supplementary Materials Tables.

## Supplementary Materials

The following are available online at https://www.mdpi.com/article/10.3390/ani11123550/s1, Table S1: Information (sex, weight and approximate age) on koalas, Table S2: Change in heart rate (bpm) after fentanyl administered as a single intravenous injection (5 mg/kg). Table S3: Plasma fentanyl concentrations over time in each koala when administered as a single intravenous bolus.
